# External validation and revision of the Lafontaine criteria for unstable distal radius fractures: a retrospective study

**DOI:** 10.1186/s13018-025-05558-w

**Published:** 2025-02-07

**Authors:** Pornpanit Dissaneewate, Phatklao Thanavirun, Yanin Tangjaroenpaisan, Kantapon Dissaneewate

**Affiliations:** 1 Department of Orthopedics, Faculty of Medicine, Prince of Songkla University, Hat Yai, 90110 Songkhla Thailand; 2 Department of Clinical Research and Medical Data Science, Faculty of Medicine, Prince of Songkla University, Hat Yai, 90110 Songkhla Thailand

**Keywords:** Distal radius fracture, Unstable fractures, Conservative treatment, Prediction rule

## Abstract

**Background:**

The Lafontaine criteria are the most commonly cited criteria for predicting unstable distal radius fractures. However, formal validation of the performance of these criteria remains limited. Therefore, we aimed to evaluate the Lafontaine criteria as a diagnostic prediction rule for distal radius fractures, assess the inter-rater reliability and predictive ability of various parameters for distal radius instability, and develop new criteria for fracture instability using reliable and highly predictive factors.

**Methods:**

This retrospective study included 274 adult patients with acute distal radius fractures treated with closed reduction and immobilisation between January 2019 and December 2022. Patients who underwent immediate surgery, were lost to follow-up before 4 weeks, or had unacceptable alignment after reduction were excluded. The Lafontaine criteria were validated using the area under the receiver operating characteristic curve (AUROC). Criteria with an AUROC > 0.7 were considered acceptable. The criteria were updated using risk factors with stronger associations in the multivariable logistic regression analysis, and the inter-rater reliability of potential predictors was evaluated.

**Results:**

The median age of the patients was 63 years; 78% were female. Redisplacement occurred in 39% of the cases. The AUROC for the Lafontaine criteria was 0.65 (95% confidence interval [CI] 0.57–0.74). Multivariable logistic regression showed that age 56–74 years (odds ratio [OR] 3.92, 95% CI 1.82–9.16, *p* < 0.001) age > 74 years (OR 6.34, 95% CI 2.66–16.2, *p* < 0.001), associated ulna fracture (OR 1.61, 95% CI 0.92–2.84, *p* = 0.10), and initial radial shortening > 3 mm (OR 5.78, 95% CI 3.11–11.2, *p* < 0.001) were the strongest predictive factors of fracture instability. These predictors demonstrated substantial inter-rater reliability, making them suitable for clinical use. Updating the model with these risk factors resulted in an AUROC of 0.74 (95% CI 0.66–0.82).

**Conclusions:**

The performance of the Lafontaine criteria in discriminating unstable distal radius fractures was unacceptable in our study cohort. The updated criteria using age group (< 56 years, 56–74 years, and > 74 years), associated ulnar fractures, and initial radial shortening > 3 mm was found to have moderate discrimination; however, further research is warranted to improve the prediction and measurement reliability of fracture instability.

## Background

Distal radius fractures are one of the most common orthopaedic injuries, accounting for up to 20% of all fractures treated in emergency departments and orthopaedic clinics [1, 2]. In adult patients, treatment strategy depends on the stability of the fracture, as assessed using a combination of fracture characteristics and patient demographics.

An unstable distal radius fracture is commonly described as one that undergoes secondary displacement following adequate reduction; however, there is no universally accepted definition of instability. Various criteria have been proposed to predict instability, with the Lafontaine criteria being among the most widely cited definitions of instability [3]. Developed in 1989, these criteria identify five predictors of secondary displacement in distal radius fractures: dorsal angulation > 20° from the initial lateral radiograph, dorsal comminution, intra-articular radiocarpal fracture, associated ulnar fracture, and age > 60 years. Lafontaine et al. [4] recommended close radiographic follow-up or surgical treatment for patients who meet at least three of these five criteria. Although the Lafontaine criteria are frequently referenced in clinical decision-making, their predictive accuracy remains a subject of debate, with limited validation studies yielding conflicting results [5–8].

Since the inception of the Lafontaine criteria, numerous new radiographic parameters predictive of fracture instability have been studied, such as the presence of a volar hook, the effective radiolunate flexion (ERLF) angle, and the metaphyseal collapse ratio (MCR) [5, 9–11]. A meta-analysis by Walenkamp et al. revealed that with the introduction of these new predictors, some of the original predictors in the Lafontaine criteria may no longer be relevant [11]. However, these new parameters have not been thoroughly evaluated for their reliability, such as inter-rater reliability, raising concerns regarding their accuracy when used by different clinicians. The advent of new predictors and uncertainty in the predictive performance of older criteria highlight the need for more accurate and reliable criteria to guide clinical decisions.

The aim of this study was to assess the inter-rater reliability and predictive value of various parameters for distal radius fracture instability, evaluate the Lafontaine criteria as a diagnostic prediction rule, and develop new criteria using more reliable and clinically relevant predictors.

## Methods

### Source of data and participants

This retrospective cohort study was conducted in accordance with the TRIPOD guidelines for clinical prediction models [12]. We included adult patients with acute distal radius fractures treated with closed reduction and immobilization between January 2019 and December 2022 at a single university hospital. Patients were identified using ICD-10 codes and matched with wrist radiographs from the orthopedic outpatient clinic and emergency department. Exclusion criteria included incomplete follow-up (≥ 4 weeks), missing initial radiographs, partial articular or radial styloid avulsion fractures, unacceptable post-reduction alignment (articular step-off > 2 mm, radial shortening > 3 mm, dorsal angulation > 0°), and immediate operative treatment.

All fractures underwent closed reduction using a finger trap with downward traction for 5–10 min, followed by manual reduction and splinting in a sugar-tong splint. Patients were followed up by an orthopedic hand specialist within one week, with splint conversion to a short-arm cast at 1–2 weeks. Immobilization lasted 4–8 weeks, with radiographs obtained before and after reduction, as well as during follow-up. For analysis, only follow-up radiographs taken at ≥ 4 weeks were included.

### Outcome measures and predictors

An unstable fracture was defined as one with follow-up radiographs showing articular step-off > 2 mm, radial shortening > 3 mm, or dorsal angulation > 10°. These criteria align with operative indications suggested by the American Academy of Orthopaedic Surgeons guidelines [8].

The evaluated predictors of instability included the five Lafontaine criteria: age > 60 years, intra-articular fracture, associated ulnar fracture (ulnar styloid or ulnar neck fracture), initial dorsal angulation > 20°, and dorsal cortex comminution. Additional predictors included the 2018 AO/OTA fracture classification [13], volar hook on post-reduction radiographs [5], and radiographic parameters measured pre- and post-reduction: articular step-off, radial height, radial shortening, radial inclination, dorsal angulation, and dorsal shift (posterior displacement of the distal fracture fragment relative to the proximal radius on lateral radiographs) following Kreder et al. [14]. The parallel method, using the lowest point of the ulnar articular surface as a reference, was chosen for radial height and inclination due to its superior interobserver reliability over the central reference point method [15]. Two additional post-reduction radiographic parameters, the ERLF angle > 25° [9] and MCR [10], were also assessed. Categorical predictors were evaluated by PD, while radiographic parameters were measured independently by KD and PD, with the average of both used for analysis. For inter-rater reliability assessment, four authors independently evaluated 50 randomly selected cases for each predictor.

### Statistical analysis

Inter-rater reliability was assessed using Fleiss’ kappa for categorical variables and ICC for continuous variables. Sample size calculations targeted 30–50 cases for reliability assessment [16, 17]. Predictors with Fleiss’ kappa or ICC > 0.5 and univariable p-values < 0.2 were included in a multivariable logistic regression using backward stepwise selection with the Akaike information criterion (AIC).

For Lafontaine criteria validation, a sample of 300 fractures was anticipated (30% instability rate), with at least 100 events and 100 non-events for a meaningful validation study [18]. Model performance was assessed using accuracy, sensitivity, specificity, area under the receiver operating characteristic curve (AUROC), and the Index of Union (IU) method for optimal cutoff estimation [19]. All performance metrics were calculated using 1,000 bootstrap resamples. Acceptable performance of diagnostic criteria for instability was set at an AUROC of ≥ 0.7 [20].

To compare redisplacement risk between the Lafontaine and new criteria, we analyzed redisplacement rates at each score level to assess the consistency of risk progression. A smoother, more predictable increase in redisplacement with higher scores was considered indicative of a more stable scoring system. Additionally, the mean radiographic Stewart score [21] was calculated at each criteria score level for pre-reduction, post-reduction, and follow-up radiographs to evaluate anatomical alignment. This analysis aimed to determine whether higher scores correlated with poorer alignment on follow-up radiographs.

The study was approved by the Ethics Committee of Prince of Songkla University (REC.65-453-11-1), with patient consent waived due to its retrospective nature.

## Results

### Characteristics of study participants

Between January 2019 and December 2022, 333 patients with acute distal radius fractures were treated at our hospital. Fifty-nine patients were excluded: 8 owing to immediate surgical treatment (4 high-demand patients and 4 with multiple fractures requiring early ambulation), 2 for partial articular fractures, 34 due to unacceptable post-reduction alignment, and 15 lost to follow-up before 4 weeks. In total, 274 patients were included in the final analysis, with a median follow-up of 53 days (interquartile range [IQR] 40–75).

The median patient age was 63 (IQR 56–74) years and 214 (78%) patients were female. One-hundred-and-eighty-seven (68%) patients had extra-articular fractures. The prevalence of each Lafontaine criterion ranged from 30 to 60%. Of 271 patients, 107 (39%) had fracture redisplacement (Table 1).

**Table 1:** Demographics and characteristics of study participants

Characteristic	*N* = 274^1^
Age	63 (56, 74)
Female	214 (78%)
Follow-up time (days)	53 (40, 75)
AO/OTA classification	
A2	83 (30%)
A3	104 (38%)
C1	36 (13%)
C2	35 (13%)
C3	16 (5.8%)
Lafontaine criteria	
Age > 60 years	169 (62%)
Intra-articular fracture	87 (32%)
Associated ulna fracture	147 (54%)
Initial dorsal angulation > 20°	83 (30%)
Dorsal comminution	125 (46%)
Volar hook	179 (65%)
Effective radiolunate flexion angle > 25 °	31 (11%)
Lafontaine criteria point	
0	19 (7%)
1	66 (24%)
2	68 (25%)
3	81 (30%)
4	34 (12%)
5	6 (2%)
Fracture redisplacement	107 (39%)

^1^ Median (interquartile range); n (%)

AO/OTA, Arbeitsgemeinschaft für Osteosynthesefragen (Association of the Study of Internal Fixation) Foundation/Orthopaedic Trauma Association

All radiographic parameter significantly improved in post-reduction x-ray from initial alignment (Table 2). The mean dorsal angulation improved from 11° pre-reduction to 4° volar angulation post-reduction. The mean radial shortening of 1.66 mm was restored to -0.13 mm in post-reduction radiograph. At follow up, there was a slight decline in alignment, with dorsal angulation increasing to a mean of 0° and radial shortening increasing from − 0.11 mm to 1.91 mm at follow-up.

**Table 2:** Initial, post-reduction, and follow-up radiographic alignment of study participants

*N* = 274	Initial	Post-reduction	Follow-up
Radial height^*1*^	5.3 (4.3)	9.3 (2.4)	6.1 (3.6)
Radial shortening^*1*^	1.7 (2.2)	-0.1 (1.4)	1.9 (1.9)
Radial inclination^*1*^	17.7 (6.2)	22.1 (3.5)	20.0 (5.3)
Dorsal angulation^*1*^	11 (16)	-4 (6)	0 (11)
Articular stepping^1^ (*n* = 87)	0.8 (0.8)	0.2 (0.3)	0.3 (0.6)
Dorsal shift^2^	1.5 (0, 3.8)	0 (0, 1.1)	-
Effective radiolunate flexion angle^2^	-	14 (8, 20)	-
Metaphyseal collapse ratio^*1*^	-	0.11 (0.12)	-

^*1*^ Mean (SD), ^2^ Median (IQR)

### Inter-rater reliability of predictors of fracture instability

The inter-rater reliability of categorical predictors varied, with the AO/OTA classification, intra-articular fracture, and dorsal comminution showing unacceptable reliability (Fleiss’ kappa: 0.36, 0.47, and 0.48, respectively). In contrast, associated ulnar fracture (0.82) and restoration of the volar hook (0.75) demonstrated good to excellent reliability (Table 3).

**Table 3:** Inter-rater reliability of distal radius fracture characteristics and radiographic parameter measurement

Fracture characteristic	Inter-rater reliability		
	Fleiss’ kappa	95% CI	*p*-value
AO/OTA Classification	0.36	0.27–0.47	< 0.001
Intra-articular fracture	0.47	0.29–0.64	< 0.001
Associated ulna fracture	**0.82**	0.70–0.92	< 0.001
Dorsal comminution	0.48	0.32–0.63	< 0.001
Volar hook	**0.75**	0.65–0.83	< 0.001

Radiographic measurement	Inter-rater reliability		
	ICC	95% CI	*p*-value
Initial radiograph measurement			
Articular step	0.42	0.28–0.57	< 0.001
Radial height	**0.97**	0.95–0.98	< 0.001
Radial shortening	**0.97**	0.95–0.98	< 0.001
Radial inclination	**0.92**	0.88–0.95	< 0.001
Dorsal angulation	**0.97**	0.95–0.98	< 0.001
Dorsal shift	**0.88**	0.83–0.93	< 0.001
Post-reduction measurement with splint			
Articular step	0.11	-0.01–0.27	0.033
Radial height	**0.89**	0.84–0.93	< 0.001
Radial shortening	**0.80**	0.72–0.87	< 0.001
Radial inclination	**0.71**	0.60–0.80	< 0.001
Dorsal angulation	**0.75**	0.65–0.84	< 0.001
Dorsal shift	**0.65**	0.52–0.76	< 0.001
Effective radiolunate flexion	0.48	0.34–0.63	< 0.001
Metaphyseal collapse ratio	0.14	0.01–0.30	0.015

CI, confidence interval; AO/OTA, Arbeitsgemeinschaft für Osteosynthesefragen (Association of the Study of Internal Fixation) Foundation/Orthopaedic Trauma Association; ICC, intraclass correlation coefficient

Inter-rater reliability varied among radiographic parameters. Most initial measurements demonstrated moderate to excellent reliability, except for articular stepping, which had an ICC of 0.42 on initial radiography and dropped to 0.11 post-reduction, indicating poor reliability. Overall, post-reduction measurements were less reliable than initial radiographs. Radial height, radial shortening, radial inclination, dorsal angulation, and dorsal shift retained moderate to excellent reliability across both time points; however, the ERLF (0.48) and MCR (0.14) had poor reliability (Table 3).

### Logistic regression analysis of predictors of fracture instability

The results of the logistic regression analysis are presented in Table 4. Among the original Lafontaine criteria, associated ulnar fracture (OR 2.07, 95% CI 1.26–3.43, *p* = 0.004), dorsal comminution (OR 2.76, 95% CI 1.68–4.58, *p* < 0.001), age > 60 years (OR 2.25, 95% CI 1.34–3.83, *p* = 0.002), and initial dorsal angulation > 20° (OR 1.72, 95% CI 1.02–2.91, *p* = 0.042) were significantly associated with redisplacement, while intra-articular fractures were not (*p* = 0.43). Additional predictors included initial radial shortening > 3 mm (OR 6.21, 95% CI 3.45–11.50, *p* < 0.001), initial radial inclination < 15° (OR 1.92, 95% CI 1.14–3.25, *p* = 0.014), failure to restore the volar hook (OR 1.94, 95% CI 1.17–3.24, *p* = 0.010), and MCR (OR 7.76, 95% CI 1.05–58.90, *p* = 0.045).

**Table 4:** Univariable, multivariable, and final model logistic regression analysis of predictors of fracture redisplacement

Predictors	Univariable regression			Multivariable regression			Final prediction model	
	OR	95% CI	*p*-value	OR	95% CI	*p*-value	OR	95% CI
Age	1.05	1.03–1.07	**< 0.001**					
Age > 60 years	2.25	1.34–3.83	**0.002**					
Age group								
< 56 years	-			-			-	
56–74 years	3.95	1.93–8.78	**< 0.001**	3.77	1.74–8.87	**0.001**	3.92	1.82–9.16
> 74 years	7.33	3.27–17.70	**< 0.001**	5.82	2.41–15.00	**< 0.001**	6.34	2.66–16.20
Intra-articular fracture	0.81	0.47–1.36	0.43					
Associated ulna fracture	2.07	1.26–3.43	**0.004**	1.64	0.92–2.92	0.093	1.61	0.92–2.84
Initial dorsal angulation	1.02	1.00–1.04	**0.013**					
Initial dorsal angulation > 20°	1.72	1.02–2.91	**0.042**	1.05	0.56–1.95	0.87		
Dorsal comminution	2.76	1.68–4.58	**< 0.001**					
Female	1.51	0.83–2.82	0.18	1.41	0.70–2.93	0.34		
Initial radial shortening	1.54	1.34–1.79	**< 0.001**					
Initial radial shortening > 3 mm	6.21	3.45–11.50	**< 0.001**	5.64	2.89–11.5	**< 0.001**	5.78	3.11–11.20
Initial radial inclination	0.94	0.91–0.98	**0.005**					
Initial radial inclination < 15°	1.92	1.14–3.25	**0.014**	0.87	0.45–1.67	0.68		
Failure to restore volar hook	1.94	1.17–3.24	**0.010**	1.38	0.75–2.53	0.29		
ERLF angle > 25°	0.72	0.31–1.55	0.41					
Metaphyseal collapse ratio	7.76	1.05–58.90	**0.045**					

OR, odds ratio; CI, confidence interval; ERLF, effective radiolunate flexion

Multivariable regression included predictors with acceptable inter-rater reliability and a p-value < 0.2 in univariable analysis. The final model identified age 56–74 years (OR 3.77, 95% CI 1.74–8.87, *p* = 0.001), age > 74 years (OR 5.82, 95% CI 2.41–15.00, *p* < 0.001), and initial radial shortening > 3 mm (OR 5.64, 95% CI 2.89–11.50, *p* < 0.001) as significant risk factors. Using backward stepwise regression with the AIC, the final predictive model consisted of age 56–74 years, age > 74 years, associated ulnar fracture, and initial radial shortening > 3 mm.

### Evaluation of the Lafontaine and new criteria for unstable distal radius fracture

We compared the discriminative performance of the Lafontaine and new criteria for predicting instability in distal radius fractures. The AUROC for the Lafontaine criteria was 0.65 (95% CI 0.57–0.74), while the new criteria demonstrated improved performance with an AUROC of 0.74 (95% CI 0.66–0.82). The optimal cutoff for the Lafontaine criteria was ≥ 3 points (accuracy 0.60, sensitivity 0.55, specificity 0.63), while the new criteria performed best at ≥ 2 points (accuracy 0.64, sensitivity 0.76, specificity 0.57) (Table 5).

**Table 5:** Performance of the Lafontaine and updated criteria for predicting fracture redisplacement

Cutoff	Accuracy^1^	Sensitivity^1^	Specificity^1^	IU^1^	AUROC^1^
Lafontaine criteria^*2*^					0.65 (0.57–0.74)
≥ 1	0.46 (0.38–0.54)	1.00 (1.00–1.00)	0.11 (0.05–0.18)	0.89	
≥ 2	0.57 (0.49–0.65)	0.83 (0.74–0.92)	0.40 (0.30–0.51)	0.43	
≥ 3	0.60 (0.52–0.68)	0.55 (0.43–0.67)	0.63 (0.53–0.73)	0.12	
≥ 4	0.64 (0.57–0.72)	0.23 (0.12–0.34)	0.91 (0.85–0.97)	0.68	
5	0.61 (0.53–0.69)	0.03 (-0.01–0.07)	0.98 (0.95–1.01)	0.95	
Updated criteria^*3*^					0.74 (0.66–0.82)
≥ 1	0.49 (0.41–0.57)	0.98 (0.95–1.02)	0.17 (0.10–0.25)	0.81	
≥ 2	0.64 (0.56–0.72)	0.76 (0.65–0.87)	0.57 (0.46–0.67)	0.19	
≥ 3	0.72 (0.65–0.79)	0.52 (0.39–0.65)	0.86 (0.79–0.93)	0.34	
4	0.64 (0.56–0.71)	0.09 (0.02–0.17)	0.99 (0.97–1.01)	0.90	

^*1*^ Mean (95% confidence interval); ^*2*^ Lafontaine criteria: age > 60 years, intra-articular fracture, associated ulna fracture, initial dorsal angulation > 20°, and dorsal comminution; ^*3*^ Updated criteria: age 56–74 years (1 point), age > 74 years (2 points), associated ulna fracture, and initial radial shortening > 3 mm, IU, Index of Union; AUROC, Area under the receiver operating characteristic curve

**Fig. 1:**
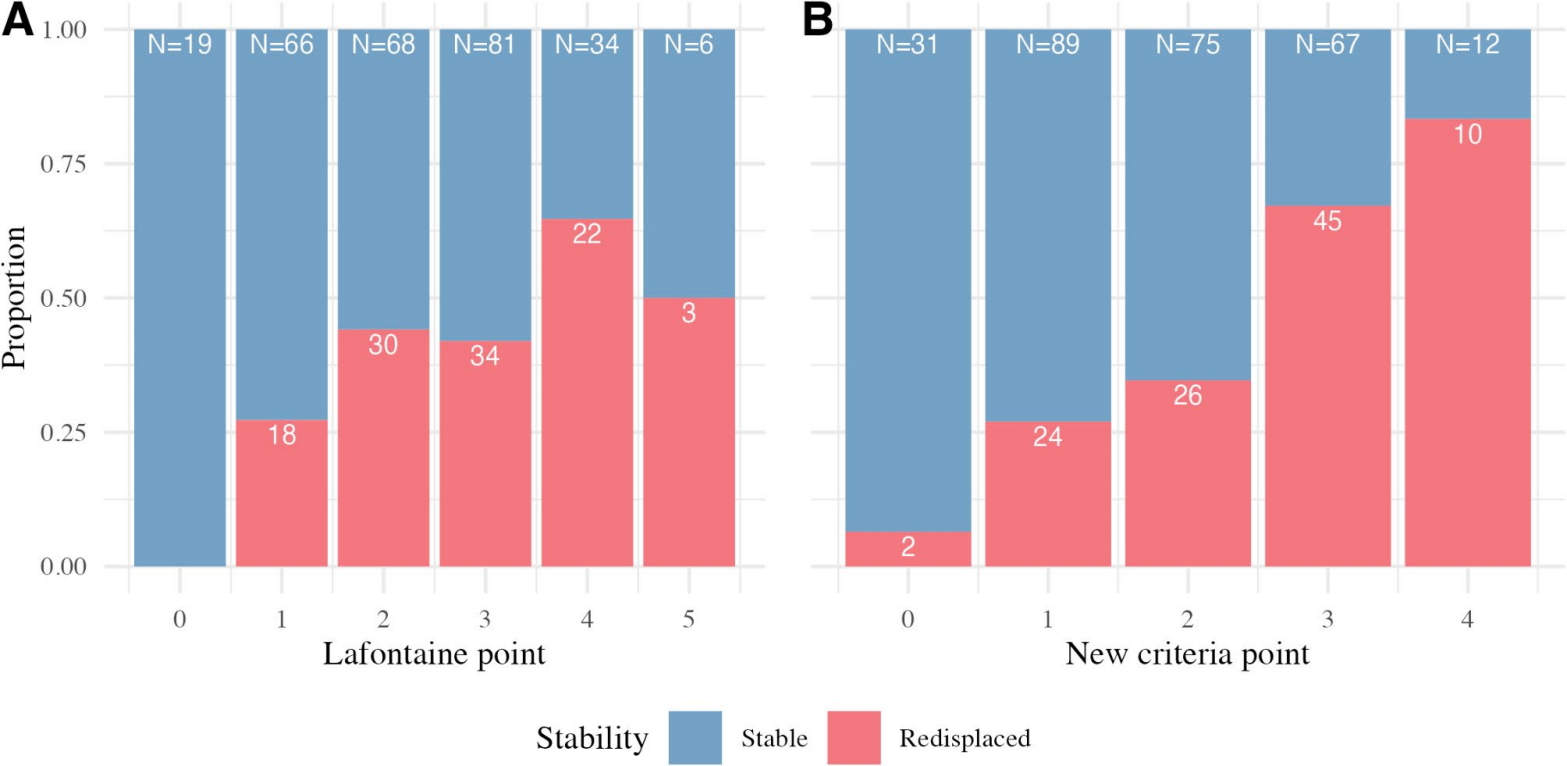
Proportion of redisplacement using the Lafontaine criteria (**a**) and the proposed new criteria (**b**) at each point. The blue bars represent stable cases, while the red bars represent cases with redisplacement. The number of cases (N) at each point is indicated at the top of each bar

**Fig. 2:**
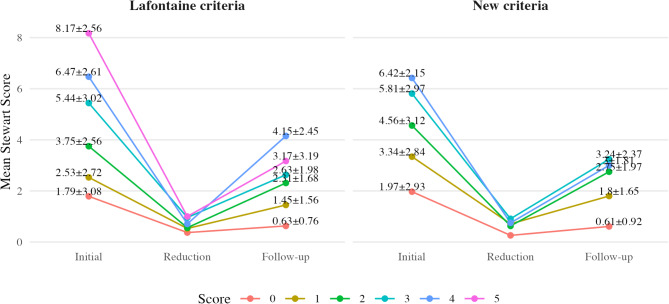
Mean radiographic Stewart score of distal radius fracture alignment in initial radiography, post-reduction radiography, and follow-up radiography, grouped by their Lafontaine criteria score (**a**) and by the proposed new criteria (**b**)

Redisplacement rates increased more consistently with higher scores in the new criteria, whereas the Lafontaine criteria showed fluctuations, particularly at higher scores (Fig. 1). This suggests that the new criteria provide a more stable progression of redisplacement risk, aligning better with clinical expectations. Similarly, higher scores in the new criteria correlated more consistently with poorer post-reduction alignment than the Lafontaine criteria (Fig. 2).

## Discussion

The Lafontaine criteria did not achieve the acceptable AUROC threshold of 0.7 in predicting fracture instability in our study. Of the original five predictors, age and associated ulnar fracture remained significant predictors, while initial dorsal angulation > 20° and intra-articular fractures were not associated with redisplacement. Dorsal comminution, though strongly associated with instability, had poor inter-rater reliability, limiting its clinical utility.

Lafontaine et al. [4] established that fractures with three or more instability factors were at a high risk of secondary displacement, and they recommended closed follow-up radiographic evaluation or surgical treatment for such fractures. While not originally intended as a diagnostic tool, Walenkamp et al. [3] found the Lafontaine criteria to be the most commonly used evidence-based definitions of unstable radius fractures in publications. Although immediate surgery for unstable fractures is desirable to promote early recovery, using the Lafontaine criteria to define instability could lead to overtreatment with operative fixation, potentially resulting in more complications, especially in the era of volar-locking anatomical plates [22, 23]. Therefore, a new clinical prediction tool is required to accurately define unstable radius fractures.

In addition to the Lafontaine criteria, we assessed new radiographic parameters, including the volar hook, ERLF angle, and MCR. Batra et al. [9] reported that an ERLF angle > 25°, indicative of radiocarpal malalignment, was highly associated with early and late fracture redisplacement. Rhee and Kim introduced the MCR as a measure of dorsal metaphyseal comminution, finding it significantly predictive of instability with excellent inter-rater reliability based on two raters [10]. LaMartina et al. [5] identified the volar hook as a strong predictor of final volar angulation and loss of volar angulation, while Mathews et al. [24] associated poor volar cortex reduction with redisplacement and malunion at 6 weeks.

In this study, the inter-rater reliability for the ERLF angle and MCR was below an acceptable threshold, limiting their reproducibility as predictors of fracture instability. The poor reliability of the ERLF angle in our study aligns with the findings of Garcia-Elias et al. [25], who reported limited reproducibility of radiolunate angle measurements, with a standard deviation of 5.2° among seven examiners. For the MCR, conflicting evidence exists. Rhee and Kim [10] reported excellent inter-rater reliability (ICC 0.812, 95% CI 0.721–0.886); however, their results were based on measurements by the two raters who developed the method, whereas our study evaluated the ICC among four raters who were potential users of the method. We found that measuring the width of the metaphyseal void on post-reduction radiographs was challenging, as reduced contrast and cast interference significantly decreased measurement reliability [26]. These factors potentially render the ERLF angle and MCR measurements unreliable.

The volar hook, or the restoration of volar cortical continuity in post-reduction radiographs, demonstrated favourable inter-rater reliability and was significantly associated with fracture instability in univariable analysis. However, its predictive value diminished when adjusted for other predictors, such as initial radial shortening, initial radial inclination, and dorsal comminution. Notably, Mathews et al. found that the volar hook was correlated with fracture redisplacement in univariable analysis; however, they did not perform multivariable analysis [24]. In contrast, our study conducted multivariable analysis and found that while the volar hook had significant predictive value in the univariable analysis, it was not significant after adjusting for other factors.

The updated criteria developed in this study included four predictors: age 56–74 years (1 point), age > 74 years (2 points), associated ulnar fracture (1 point), and initial radial shortening > 3 mm (1 point), with a score of two or more indicating instability. While associated ulnar fracture and age were part of the original Lafontaine criteria, we revised the age threshold from > 60 years into two categories based on population quartiles, allowing for better risk stratification without becoming overly complex. The addition of initial radial shortening, an established predictor of fracture instability, further highlights the relevance of initial displacement as a predictor of fracture redisplacement [27–29]. The updated criteria exceeded our target AUROC of 0.7, reflecting an improvement over the original Lafontaine criteria. However, the improvement was marginal and less substantial than anticipated, suggesting that the relationships among radiographic parameters may be too complex to be fully captured by traditional logistic regression models. Another consideration would be to include additional predictors from early follow-up x-rays, such as marginal secondary displacement at 1–2 weeks, which Schmidt et al. [30] found to be highly associated with malunion at 3 months. While this approach could lead to a longer wait time before making surgical decisions, it may significantly improve the discriminative ability of the criteria. Unfortunately, we do not routinely perform follow-up x-rays in the first week and therefore could not evaluate this predictor.

We applied stricter criteria for post-reduction alignment, specifically excluding fractures with dorsal angulation > 0°, to reduce the risk of redisplacement. Accepting a dorsal angulation of < 10° immediately after reduction could mean that fractures with an angulation close to 10°, such as 9°, would be considered acceptable. However, even a small collapse of 1–2° at follow-up would quickly classify these fractures as unacceptable, increasing the likelihood of redisplacement. Given the likelihood of an additional 5° of dorsal angulation in follow-up x-rays within the first week [30], our decision to use more stringent alignment criteria at the post-reduction stage is justified to better identify fractures at risk for redisplacement.

This study had several notable strengths. To our knowledge, it is the first to formally validate the performance of the Lafontaine criteria in predicting unstable distal radius fractures. Additionally, we utilised both inter-rater reliability and the strength of association to determine the suitability of the predictors for identifying unstable fractures.

However, this study had some limitations. Its retrospective nature may introduce selection bias, though the use of radiographic parameters minimizes this risk. One major limitation was the follow-up period of at least 4 weeks rather than the 6-week minimum used in some studies, which may have led to an underestimation of late redisplacement cases. Additionally, while our sample size was adequate for analysis, it was not large enough to assess complex interactions between radiographic predictors. This study was conducted at a single center, which may limit the generalizability of our findings. Moreover, we only evaluated radiological outcomes, which may not fully capture the differing functional outcomes based on a patient’s functional demands. Patients with higher functional demands, despite older chronological age, may benefit more from our criteria for acceptable alignment than those with lower functional demands [31, 32]. Future studies should validate these criteria in larger, multicenter cohorts with longer follow-up periods to enhance robustness and external validity.

## Conclusions

The Lafontaine criteria demonstrated unacceptable discriminative performance in predicting unstable distal radius fractures. Although the updated criteria showed an improved performance, there is still room for further enhancement. The reliable measurement of radiographic parameters is essential for accurately evaluating the risk of redisplacement. Future studies should focus on validating these findings across diverse patient populations and clinical settings to establish more robust criteria for predicting redisplacement.

## Data Availability

The datasets used in this study and the codes used to produce the findings of this study are available from the corresponding author upon request.

## Abbreviations

AIC: Akaike information criterion
AO/OTA: Arbeitsgemeinschaft für Osteosynthesefragen (Association of the Study of Internal Fixation) Foundation/Orthopaedic Trauma Association
AUROC: Area under the receiver operating characteristic curve
CI: Confidence interval
ERLF: Effective radiolunate flexion
ICC: Intraclass correlation coefficient
IU: Index of Union
MCR: Metaphyseal collapse ratio
OR: Odds ratio
